# A multiple case study of pre-diabetes care undertaken by general practice in Aotearoa/New Zealand: de-incentivised and de-prioritised work

**DOI:** 10.1186/s12875-023-02053-1

**Published:** 2023-04-29

**Authors:** Christine Barthow, Jeremy Krebs, Eileen McKinlay

**Affiliations:** 1 Department of Medicine, University of Otago, PO Box 7343, Wellington, Wellington South 6242 New Zealand; 2 Department of Primary Health Care & General Practice, University of Otago, PO Box 7343, Wellington, Wellington South 6242 New Zealand

**Keywords:** Pre-diabetes, General practice, Health promotion, Primary prevention, Health equity, Qualitative research, Organisational case studies, Models of care, Interdisciplinary, Case study

## Abstract

**Background:**

In Aotearoa/New Zealand (NZ) general practices diagnose and manage pre-diabetes. This work is important as it has the potential to delay or prevent the onset of Type 2 Diabetes (T2DM), reduce NZ’s health inequities, and the burden that T2DM places on health care services. However, no study has previously examined how this work routinely occurs in NZ.

**Methods:**

Two case studies of practices serving ethnically and socio-economically diverse populations, followed by cross-case analysis.

**Results:**

The NZ health care context including funding mechanisms, reporting targets, and the disease centred focus of care, acted together to dis-incentivise and de-prioritise pre-diabetes care in general practices. The social determinants of health differentially influenced patients’ ability to engage with and respond to pre-diabetes care, significantly impacting this work. Differing perspectives about the significance of pre-diabetes and gaps in systematic screening practices were identified. Interventions used were inconsistent and lacked comprehensive ongoing support.

**Conclusions:**

Complex multi-layered factors impact on pre-diabetes care, and many of the barriers cannot be addressed at the general practice level. The practice serving the most disadvantaged population who concurrently have higher rates of pre-diabetes/T2DM were more adversely affected by the barriers identified.

**Supplementary Information:**

The online version contains supplementary material available at 10.1186/s12875-023-02053-1.

## Background

Diabetes mellitus is a growing global health and economic emergency [1], and ranks amongst the top ten causes of death worldwide [2]. In 2021, the International Diabetes Federation estimated that almost 537 million people (aged between 20–79) had diabetes and predicted this will increase 46% by 2045 [3]. Approximately 90% of those with diabetes will have Type 2 diabetes mellitus (T2DM). Diabetes occurs more frequently in urban compared to rural areas [3], and disproportionately affects indigenous and disadvantaged populations [4, 5].

Pre-diabetes, where blood sugar levels are elevated but are not high enough to be classified as T2DM is considered a high-risk state for the development of diabetes [6, 7]. Internationally diagnostic criteria for pre-diabetes are inconsistent; however, pre-diabetes affects large numbers of adults and increasing proportions of younger populations. In England, 35% of those aged over 16 years had pre-diabetes, and 50% of those aged over 40 years with body mass index (BMI) > 25 had pre-diabetes in 2011 [8]. Within Aotearoa/NZ (NZ), outdated estimates are that 25% of adults have prediabetes [9]. 

Pre-diabetes is asymptomatic and for many the diagnosis is unexpected; however, its diagnosis provides an important opportunity to prevent disease. Effective interventions, particularly lifestyle interventions to reduce weight, modify diet and increase physical activity can reduce hyperglycaemia and prevent or delay the onset of T2DM [10–14]. Metformin may also be helpful; however, it is less effective than intensive lifestyle change and effects may vary according to subgroups [15–18]. Internationally the diagnosis of pre-diabetes predominantly occurs in primary care, however the responsibility for delivery of lifestyle interventions varies. In some jurisdictions including the United States, United Kingdom, Australia and Finland, people with pre-diabetes are offered the opportunity to attend free intensive community-based and separately funded diabetes prevention programmes [19, 20]. In contrast, NZ has a less structured approach predominantly relying on primary care to do this work by educating patients about pre-diabetes, promoting lifestyle changes, monitoring effects of the interventions and prescribing metformin if needed. (See Table 1 for details of how NZ general practice provision is funded).

**Table 1:** Description of NZ general practice funding models

General practices in NZ
NZ general practices operate using a mixed model of publicly and privately funded health care; however, this funding arrangement contributes to inequities [26]. Individuals formally enrol with a specific general practice and generally each time they see a staff member (typically general practitioners (GPs) and primary care nurses, but increasingly other health support workers), they make a co-payment. Practices receive government capitation funding which accounts for population demographics with funding-payments made according to the number of people enrolled and not the number of times a provider sees each patient. Practices serving populations in which at least 50% are classified as high needs (e.g. Māori, Pacific or lower socioeconomic) may choose to operate as Very Low-cost Access (VLCA) practices. In return for additional government funding these practices provide free services for children 13 years or younger and, and maximum co-payments for all other age groups are set at low levels [26]. (For example, see fee comparisons in Table 3.) This is intended to support practices to develop service delivery models that are most suited to the populations they serve and reduce health inequities

NZ is internationally considered to have a strong primary care system [21]. Yet primary care has been recognised not to meet the needs of Māori, Pacific Peoples and those on low incomes [22–24] and upcoming health reforms will attempt to address this [25]. 

As the majority of pre-diabetes care in NZ is provided by general practices it is important to understand how this work is currently conducted, and to date this has not been examined within the NZ context. By learning how this care is delivered we can advance our understanding of this work and identify barriers and facilitators that may be altered to optimise the diabetes prevention and improve diabetes related health equity, and such findings are likely to have international applicability.

## Methods

### Aim

The aim of this study was to describe how pre-diabetes is detected and managed by two NZ urban general practices.

### Ethical considerations

Ethics approval for this study was granted by the University of Otago Human Research Ethics Committee (Minimal risk health research), approval number DH20/027.

### Study design

A multiple case study design including two embedded qualitative case studies with cross case comparison was used [27]. The strength of using a case study approach for this research is its ability to answer ‘how’ and ‘why’ type research questions [27, 28] and convey a multifaceted understanding of human behaviours, social interactions, processes and context, all of which interact to influence the provision of health services [29]. This understanding is achieved through multiple, flexible approaches to data collection, analysis and triangulation [27, 28]. We used a lens of appreciative inquiry, a strengths-based approach which values different world views to ensure that the experiences described by both practices were considered equally valid [30]. 

### Setting

Three urban practices were approached to take part in the research. These practices were purposively selected [31] for diversity related to the enrolled populations as we anticipated that care provision would differ according to population demographics. Two practices agreed to participate: one had a population base similar to the NZ demographic profile, while the other a Very Low-cost Access (VLCA) practice, had higher proportions of ethnic groups who have higher rates of T2DM. Each practice knew about the other practice; however, to avoid influencing the focus group discussions, very limited information was shared between the two practices.

### Data collection and analysis

**Fig. 1:**
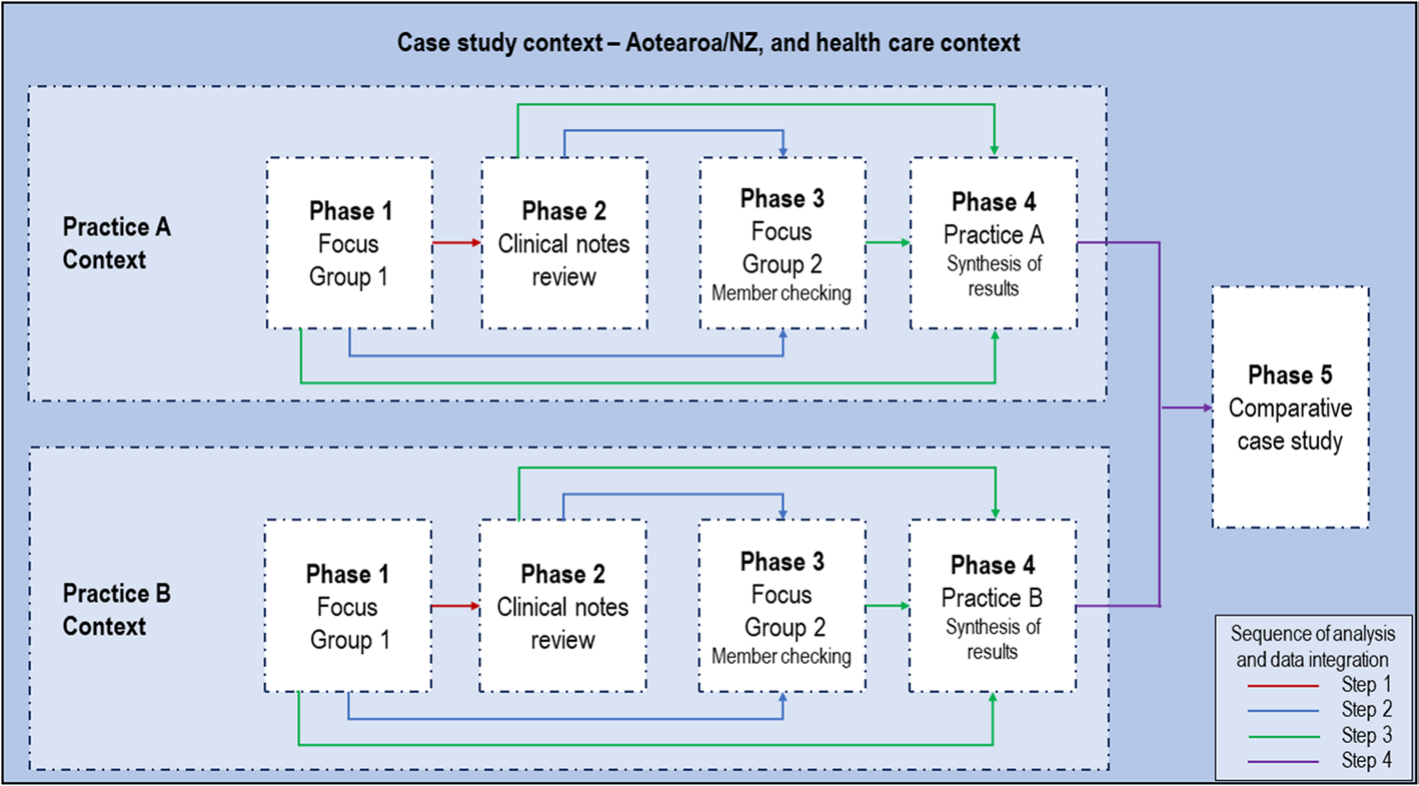
Case study design

Sequential collection and analysis of multiple sources of data [27] from each practice was undertaken, with each step informing the next (see Fig. 1). In phase one separate recorded focus groups were undertaken by CB and EM with key clinical staff from each practice, to gain a broad overview of the importance of pre-diabetes care and usual clinical practices in relation to pre-diabetes care. Phase two involved an in-depth retrospective review of purposively sampled anonymised clinical notes from each practice [32]. NZ classifies those with a glycated haemoglobin (HbA1c) 41–49 mmol/mol as having pre-diabetes. Specifically, anonymised records from individuals with HbA1c values in the higher pre-diabetes range (45–49 mmol/mol) and up to ten years HbA1c data were sought, including records from males and females, a range of ages and ethnic origins. In phase three the themes from phases one and two were reported back to each practice by CB and EM to a larger group of staff as a method of member checking [27] and to seek further responses. During phase four all practice data were integrated into a completed individual practice case summary. Finally in phase five, a cross-case analysis was performed. A study protocol, fieldnotes, recordings, transcripts, anonymised case records, and analyses were maintained to form a chain of evidence and to ensure reliability [27].

### Analysis of focus group data

Consistent with multiple case study research, data sets for both practices were analysed and interpreted separately until the final cross-case analysis [27]. All focus group data were transcribed, checked for accuracy, and coded in Microsoft Word. Initial deductive or content coding [33] was undertaken according to the focus group questions (CB) and discussed (CB, EM). It became apparent each practice had both similar and unique ways of approaching pre-diabetes care which could not be demonstrated through deductive coding alone, and inductive thematic coding [34] was undertaken, and separate lists of themes were generated.

### Analysis of clinical records

From each set of notes, data was coded and extracted into a template prior to being presented in a graphic single case review. The template was designed to ensure data collected accurately represented general practice work where: multiple health issues are frequently addressed in one visit; multiple different providers may provide care; other health monitoring and disease prevention tasks are undertaken; and to reflect care provided over time. The full sequence of case note data management is presented in Supplementary Table 1 (in Additional file 1) and two examples of completed case review summaries are presented as Supplementary Figs. 1A and 1B (in Additional file 1).

For each practice, the full set of clinical note summaries were considered together, and findings synthesised for reporting back to the practice during the second focus group.

### Cross case analysis

A final analysis was undertaken of the entire dataset (all data from each practice), and the cross-case themes were generated. These were identified by considering both cases as a whole and in particular noting key similarities and differences which shaped the different approaches to pre-diabetes care in each practice.

### Researcher roles and reflexivity

Researchers EM and CB are Pākehā (non-indigenous New Zealanders of European descent) registered nurses with academic roles and have no clinical involvement with the practices included in this study. EM is experienced in qualitative and primary care research. JK is a Pākehā doctor with both clinical and academic endocrinology roles and has regular clinical interactions with both practices, and to avoid a conflict of interest he did not attend focus groups. However, all researchers regularly discussed the evolving interpretation of the data and insights gained from the study.

## Results

Data collection occurred between June 2020 and July 2021. A summary of those attending focus groups and the number of clinical records reviewed from each practice is presented in Table 2.

**Table 2:** Summary of focus group attendees and clinical record data

	Practice A			Practice B		
	**Phase 1 Focus group**	**Phase 2 Note review**	**Phase 3 Focus group**	**Phase 1**^**c**^** Focus group**	**Phase 2 Note review**	**Phase 3 Focus group**
Clinicians						
GPs	2		5	1		9
Nurses	3		6	2		6
Allied health workers^a^	0		0	0		1
Health support workers^b^	0		1	5		4
Case notes reviewed		6			5	

GPs General practitioners ^a^ includes Pharmacist, Social worker, Dietitian ^b^ includes Health Improvement Practitioner, Health Coach, Cultural Support Worker, Community Health Worker, Health Care Assistant. ^c^ Focus group performed via video conferencing due to COVID-19 pandemic

As shown in Table 3, practice characteristics varied according to the funding models, practice workforce, demographics of populations served, and the underlying prevalence of detected pre-diabetes and T2DM.

**Table 3:** Practice characteristics

	Practice A	Practice B
Location	Urban practice, New Zealand City	Urban practice, New Zealand City
Region decile scores^a^	1–5 (least—medium deprivation)	10 (most deprived)
Enrolled population^b^		
Total	12,500	7000
Māori	10%	23%
Pacific Peoples	5%	46%
European and other	85%	31%
Business model	Privately owned	Incorporated Society
Funding model	Capitation formula	Very low-cost access
Practice fees^b^		
Under 14 years	free	free
14–17	NZ$39	free
18–24	NZ$44	free
25–64	NZ$50	NZ$18
Over 65	NZ$47.50	NZ$7
Nurse consultation	NZ$25–30	free
Staffing mix	GPs, nurses, social worker, pharmacist, health care assistant	GPs, nurses, social worker, pharmacist, health support workers^c^
Staff directly involved in pre-diabetes care	GPs, nurses. Referral to other services as required	GPs, nurses, health support workers. Referral to other services as required
Proportions of enrolled population with pre-diabetes or T2DM^b^		
Pre-diabetes or T2DM	8%, *n* = 961	16%, *n* = 1101
Pre-diabetes		
Total	3.1%, *n* = 491	8.7%, *n* = 559
Māori	3.2%	7.5%
Pacific Peoples	6.3%	10.2%
T2DM		
Total	3.8%, *n* = 470	7.3%, *n* = 502
Māori	3.9%	5.1%,
Pacific Peoples	8.6%	9.7%,

*GPs* General practitioners, *T2DM* Type 2 diabetes Mellitus. ^a^Deprivation scores https://www.ehinz.ac.nz/indicators/population-vulnerability/socioeconomic-deprivation-profile/ accessed 21 May 2021. Interpretation of NZ index of deprivation scores – 1 is least deprived, 10 is most deprived. ^b^ Data provided by practices. ^c^ Health support workers – includes Cultural support worker, Health improvement practitioner, Health Coach, Health care assistant, Community health workers

### Case specific themes

The case specific themes generated from the independent analysis of each case study are summarised in Table 4.

**Table 4:** Case specific themes

Practice A	Practice B
**1. Perceptions of Pre-diabetes** Pre-diabetes is an ambiguous condition with uncertain outcomes The diagnosis of pre-diabetes provides a Health coaching/Educational opportunity Pre-diabetes is a social issue	**1. Team based approach and model of care** A team-based approach is critical to pre-diabetes care
**2. Change facilitators** Good clinician – patient relationships are critical Change is gradual, a L longitudinal approach is required	**2. Diabetes prevention work** Complex care, hard to prioritise Time consuming Limited by system level/societal issues
**3. Challenges** Who to target and how intensively? How can pre-diabetes care be targeted to those most at risk and to ensure the best use of resources/time? Competing clinical priorities Patient readiness to change Weight loss vs other approaches Practice record systems	**3. Change facilitators** Engagement, motivation, personal agency Acceptable intervention options
	**4. Challenges** Concentration of high needs population Social determinants of health Health literacy Normalisation of diabetes Vulnerable missing groups

Themes are noted in bold with subthemes underneath

### Cross case findings

Four themes were developed from the cross-case analysis. Two themes concern contextual factors, and two themes relate to participants’ perspectives and current clinical practices. The four themes are: 1. health care context, 2. practice population and the social determinants of health, 3. perspectives regarding pre-diabetes, and 4. current practices.

#### Theme 1: Health Care Context

Both practices noted that NZ’s primary care funding is linked to reporting targets. They pointed out that while targets for smoking cessation and T2DM care exist, there are no targets, and consequently no specific government funding available to support pre-diabetes care. The impact was particularly evident in Practice B which had a twofold higher proportion of people with pre-diabetes and T2DM in their enrolled population than Practice A (Table 3). This exacerbated workload and funding issues, as the higher proportions of those with T2DM resulted in an* ‘exponential increase in workload, not a linear increase in workload*’ *(Practice B GP 2),* which was not accounted for at a policy level by increased practice resourcing.

… [it’s] our targets that really drives a lot of what we do and what we prioritise, and if it’s over 65’s then it’s over 65’s, so what happens to the others? … so our funding is connected to [targets], so we’re always aiming to get that. (Practice B HCW 3).

Co-payments, even though set at a lower level for Practice B (Table 3) were described by Practice B GP 2 as ‘*a fundamental flaw in the system*’ and were noted to deter patients, particularly those who were on the ‘*breadline*’, from proactively seeking care for conditions like pre-diabetes.

Both practices felt the combination of funding model constraints, and the broad scope of general practice work resulted in the de-prioritisation of pre-diabetes care. Unlike specialist services focusing on single disease entities, the fifteen-minute GP consultation is generalist, and necessitates clinical prioritisation of the most urgent health needs, and is often focused on established disease, complex multimorbidity or acute and urgent presentations rather than promoting longer term health through pre-diabetes care.

There is an issue, where you’re always prioritising what you’re dealing with, ‘cause you can’t deal with everything… And pre-diabetes, I think just drops right down the list, often, because the other issues are more pressing. … it’s really, really difficult to create space for pre-diabetes. (Practice B GP 2).

These findings were confirmed and extended by the clinical notes review. Patients with established long-term conditions attended general practice more frequently however the opportunity to provide pre-diabetes care was often not utilised or received only minor attention due to the pressures of other health priorities and short appointment time. In contrast, those without co-morbidities attended general practice less frequently and therefore were not available to receive opportunistic pre-diabetes care.

#### Theme 2: Practice population and the social determinants of health

Both practices described the impact of the wider social determinants of health (SDOH) on pre-diabetes, and the limitations of the health system to address these issues.

Because some of it’s economic. You know, it’s social. It’s about having the right job, … and being able to afford to buy veggies as opposed to bread at $1 a packet. … It’s not just health providers that make a difference (Practice A GP 1).

Practice B situated in the lowest decile area (Table 3) identified multi-layered consequences related to social deprivation. Firstly, as demonstrated below, low socio-economic status compromised individuals’ ability to prioritise health:

… the rent prices going up, especially with this community, where they’re in emergency housing, and they’re not able to take time off work…. Like these are the people [for whom their health] isn’t their first priority and usually they’re not their first priority, their children are, or their family members are. (Practice B Nurse 2).

Secondly, finding time or physically accessing general practice appointments was extremely difficult for this group particularly those in unstable, casual, or unsupportive employment conditions. Additionally, logistical issues such as ‘*there’s no money for petrol, and there’s no car. They have a lot of young children that they can’t take on the bus’ (Practice B GP 1)* were barriers to attendance.

Finally, even when patients were able to attend appointments and receive relevant lifestyle advice, competing demands for limited financial resources meant patients did not always have the additional funds to follow the recommendations.

I was talking to a patient about … diet, and he says I’ve got four children, and they’re not going to eat what you want me to eat, so I’m not going to take my money to buy food that nobody’s going to eat. … he said at the end of the day, we’re still struggling just to pay for rent. (Practice B Nurse 3).

These two higher level themes: the health care context and practice populations, appeared to directly impact on each practices’ attitudes to pre-diabetes care and how this work was undertaken in each practice. These are discussed next.

#### Theme 3: Perspectives regarding pre-diabetes

There were differences between practices perspectives regarding pre-diabetes. In Practice A some GPs and particularly the nurses, perceived that the diagnosis of pre-diabetes provided an opportunity to educate patients and encourage healthier lifestyles: ‘*[start] early and make those changes sooner’ (Practice A Nurse 1).*

GPs acknowledged the imprecision of HbA1c measures and that the wide range (41–49 mmol/mol) used in NZ for diagnosis [35] classifies a very large group of individuals as having pre-diabetes. Furthermore, they observed that many did not later develop T2DM. As illustrated below this prompted concern about poorly targeted use of health care resources for little anticipated gain.

I think we invest a huge amount of time in people with HbA1c at 41, who are never going to get diabetes. I’ve tracked these people for 20 years, and they don’t. Whereas if you’ve got an HbA1c of 46, 47 and you’re obese, and you’re Indian, Pacific Islander, [have] a family history [of diabetes], then you’re probably going to get diabetes, (Practice A GP 1).

Based on their clinical observations, and to manage workloads these clinicians had informal ways of assessing the level of risk posed to an individual which often included watchful monitoring of HbA1c and BMI trends over time. It appeared that those who had HbA1c’s in the lower pre-diabetes range were predominantly informed of the diagnosis, given written information, but with no detailed discussion, and then simply scheduled for future retesting. In contrast staff at Practice B appeared to be concerned about HbA1c at any point in the pre-diabetes range however the extent to which pre-diabetes was targeted for intervention was partly reliant on whether the patient was engaged: ‘*If they’re engaged, then you just really try and target them. It doesn’t matter what their HbA1c is, … 41 or 48’ (Practice B GP 5).* This difference in attitude may reflect the influence of the high prevalence of disease and the socio-economic barriers encountered when working with this population.

#### Theme 4: Current practices

##### Detecting pre-diabetes

Screening for pre-diabetes is largely conducted as part of the NZ cardiovascular disease risk assessment programme linked to age and ethnicity [36], however screening in younger groups is also recommended when two or more prespecified risk factors are identified [35]. Practices identified groups such as men, or women with a history of gestational diabetes who were less likely to attend general practice and might miss screening. Additionally, Practice B was concerned about the rising rates of pre-diabetes in younger groups and that these groups were less likely to have their pre-diabetes detected.

##### Different models of care and ways of working

Both practices operated using distinctly different staffing mixes and roles (Table 3). Practice A only utilised GPs and nurses to provide pre-diabetes care with occasional referral to Green Prescription providers [37] and community exercise groups. Some GPs managed those with pre-diabetes alone. Clinicians in this practice discussed a variety of approaches to pre-diabetes care. Education regarding pre-diabetes appeared to focus on biomedical concepts and the risk of progression to diabetes. Visual tools were used to facilitate patients’ understanding of their HbA1c levels, and sometimes serialised weight and HbA1c levels were shown to help patients see the relationship between weight and HbA1c. Dietary advice was tailored to include cultural food preferences, and identification of specific actions such as ‘*not having a packet of chips every night’ (Practice A Nurse 2)* were discussed*.* Nurses occasionally described that if the patient was motivated to make changes, they would get patients to return to assess progress with the anticipation that ‘*coming in and engaging with the actual act of doing that, then that that could keep them on track*’ *(Practice A Nurse 3).* However, limited follow-up support was provided by either GPs or nurses and overall, it appeared that the underlying pattern of care was:

… all about just trying to give them the best information in the time you have in a way they understand, and ultimately, it’s their responsibility to do with that what they will. (Practice A Nurse 2).

Practice B employed a range of health support workers in addition to clinicians. (See Table 3) Depending on the circumstances any of these support staff could be involved in pre-diabetes care ‘*so with health promotion here, we use everyone’ (Practice B Nurse 1).* This practice had incorporated a new health support worker role (Health Improvement Practitioners (HIP) [38]) in the year before this study, and patients were not charged for their services. When patients agreed, GPs and nurses referred patients with pre-diabetes to these staff. As illustrated below, while these support workers were culturally well matched (they belonged to Pacific communities), they also encountered difficulties attributable to the SDOH.

… it’s really good that we do get the whole handover straight away from our GPs and nurses … so we get to like talk to them right there on the spot, and then just follow on from what’s been said, but you know, the diet and exercise. … the sad thing is, …. for some of them, they don’t attend. … What we’re realising now, is what the doctors have been going through. [Like the Doctors] we’re chasing them up to make sure they are trying to, or they need extra support or other programmes we can refer them to, …. A lot of people are the same, their self-care’s usually last thing, and we try and work around it as well. (Practice B HCW 1).

In this practice health literacy was recognised as very important: *‘When you talk about the [HbA1c] numbers, they don’t understand that, and they won’t know how important it is. … they only remember what they actually understood’ (Practice B Nurse 1).* As discussed by Practice B HSW 1, considerable effort was put into checking information and attempting to motivate patients to *‘get that message through, that they can actually make that change themselves’* and ‘*pushing them to try and make those changes’*. Acknowledging this difficulty, the health worker said:

Sometimes patients struggle to change behaviours and beliefs they have been doing for most of their lives. So, I try to focus on their values/beliefs and their strengths, this helps with motivation. (Practice B HSW 1).

Staff also set goals with patients regarding achievable lifestyle actions and provided regular follow-up for two to three months. This practice felt referrals to community physical activity groups were more effective if these services were well matched and integrated into their community as ‘*people find themselves more willing to work, with other people who are suffering from the same conditions*’* (Practice B HSW 1).*

Practice B also tried different approaches, including running a group session with individuals and whānau/families to hear a member of the community ‘*share their testimony’ (Practice B HSW 1)* about achieving weight loss and reversal of diabetes. For staff who lived locally, their role extended into the community.

it’s really important to also engage in the community as well, instead of just being in our clinic, … so, it’s being visible, … and you know, walking the talk… it is about walking alongside them. (Practice B Nurse 2)

##### Targeted weight loss and metformin use

Both practices experienced challenges in directly discussing the central role of weight loss in reversing pre-diabetes, and in supporting patients to lose weight. While some reported saying to patients ‘*the best thing you can do is lose weight’ (Practice A GP 4),* factors such as not wanting to ‘*dishearten patients*’ *(Practice A GP 1)* if weight did not go down, were reasons to focus on exercise and healthier eating instead. Added to these concerns were cultural sensitivities: *‘because food has a lot to do with celebrating, and … every part of our culture’ (Practice B Nurse 2).*

Patterns of metformin use differed between practices. In Practice A the general attitude was ‘*it wouldn’t cross my mind really to start metformin just on the basis of pre-diabetes*’ (*Practice A GP 2)*. While a few patients were prescribed metformin, it was only used in the context of high HbA1c levels (49 mmol/mol) and co-existing metabolic issues. In contrast, in Practice B, metformin was prescribed based on pre-diabetes alone; ‘*[HbA1c] 45 plus, I start’ (Practice B GP 2),* or *‘around 47/48 up, I often give metformin straight away*’ *(Practice B GP 5).*

##### Documentation, monitoring, and follow-up

In both practices documentation about pre-diabetes care was inconsistent. Nurses and health support workers wrote more detailed records while some GPs relied on practice management system coding to report their actions related to pre-diabetes. Others reported ‘*I do talk [to the patient] … but I forget to write, because of time pressure’ (Practice B GP 3).*

Monitoring patients was largely driven by annual recalls for blood tests, which created significant workloads and was not always successful in either practice: ‘*I will contact [patients]… three or four times, and I just give up for another year’ (Practice A GP 1)*.

Neither practice knew if their interventions for pre-diabetes did result in substantial changes in lifestyle, and evaluation did not appear to be a high priority. ‘*We don’t always know if it works, but you either plug away at it, or you just give up’ (Practice A GP 1).*

## Discussion

Internationally T2DM is a major and increasing health problem and diabetes prevention is a critical health equity issue. In NZ persistent diabetes disparities relate to ethnicity particularly for Māori, Pacific, and Asian groups [39]. Additionally, low socioeconomic status, which is frequently present in these groups, is an independent risk factor for T2DM [40]. As in many other countries pre-diabetes is predominantly diagnosed in primary care settings and in NZ after diagnosis it is largely being managed within general practice as very few specific/stand-alone pre-diabetes lifestyle programmes exist. Until now no attention has been given to how general practices in NZ undertake pre-diabetes work and whether it is the most appropriate setting or workforce to be doing this work. This case study of two diverse general practices identified multilevel, interacting factors impacting on the provision of pre-diabetes care. Case specific themes reflected the common and unique concerns of each practice. We found significant variability within and across practices in their provision of pre-diabetes care. The cross-case analysis in this study showed this variability was influenced by the health care context, and the characteristics of the practice population including the SDOH and the impact of these factors on health provider attitudes and care provision. When combined these factors de-prioritised pre-diabetes care, largely rendering it invisible.

### Findings within the context of current literature

General practice work addresses a comprehensive range of health issues including primary disease prevention. However, as has been reported internationally [41], both NZ practices struggled to prioritise pre-diabetes care as it was not supported by adequate specific funding and the workload associated with other core functions of primary care took precedence. Like many other countries, current general practice funding in NZ favours treatment of established disease, acute care, or activities such as smoking cessation. Targets linked to these activities operate as powerful disincentives for management of conditions like pre-diabetes. Additionally as pre-diabetes is asymptomatic, unless specifically raised by health professionals it is unlikely to be a priority for patients [26]. Furthermore, patient co-payments required for most services, even when reduced in VLCA practices, deter patients from proactively seeking care and regular attendance at general practice for ongoing support and review [42]. Internationally successful diabetes prevention interventions funded separately and operated outside of general practice have demonstrated that long term support is required to establish and sustain successful lifestyle change [41], and perhaps NZ and countries with similar funding systems should consider adopting such programmes. However, as has been shown internationally these programmes require cultural adaptations and may have lower uptake or benefits for the most disadvantaged and ethnic minority groups most in need of effective strategies for diabetes prevention [43–45]. 

The clinical records review showed those with pre-diabetes often had multimorbidity which increased complexity and competed with the time to address pre-diabetes. While evident in both practices, these barriers were compounded and more prominent in the practice serving a high needs population, including Māori, and Pacific populations who have a higher burden of multimorbidity [46–48]. In this practice even with an explicitly team-based model of care, for nurses and culturally matched support workers with longer consultation times than GPs, it was a struggle to make progress. This additional workload and complexity are not recognised or adequately supported by VLCA funding. Similar findings have been described in NZ [49] and internationally [50]. 

Both practices described barriers to pre-diabetes care related to the SDOH; however, these were markedly worse in Practice B. As in other counties with indigenous peoples, the SDOH, relate to colonisation and racism [51–54], are inequitably distributed and are key drivers of health inequalities. SDOH account for 50–60% of health outcomes, while health care plays a small part, influencing approximately 10–15% of outcomes [55]. Patients in Practice B had multiple disadvantages including poor health literacy, unstable housing, food insecurity, were harder to reach, and were more likely to have limited psychosocial and/or financial agency to change their situation or behaviour. These findings are consistent with international research in similar groups [56, 57]. The impacts of SDOH translated into increasing demands on Practice B, and culturally congruent staff sought to mitigate these challenges by ‘*working around’* patients’ inability to prioritise their own health while providing care. However, unsurprisingly they were not always effective, and this reflects that health systems alone cannot ameliorate social and financial problems.

In this study both practices frequently referred to levels of ‘patient engagement’. Higgins *et. al.* define patient engagement as ‘*the desire and* ***capability*** *to* ***actively choose to participate*** *in care in a way uniquely appropriate to the individual…*’ [58] p. 30. This study shows the requisites for this type of engagement are not possible for those experiencing socio-economic disadvantage. The current approaches to diabetes prevention attribute both the problem of being at risk for diabetes and the solutions, that is achieving sustained lifestyle change, to the individual, and assume they have the capacity to ‘engage’ and the personal agency to make change [41, 59, 60], and this approach is not appropriate for disadvantaged groups [57, 61]. Therefore, multisectoral approaches including public health approaches are needed to address the diabetes epidemic, and these need to address the fundamental root causes and contributors to the diabetes epidemic including racism, SDOH, poverty, obesity, food and physical environments [5, 26, 40, 47, 59, 62–68]. 

Both practices identified important gaps in screening for pre-diabetes, especially affecting groups who fall outside the inclusion criteria for cardiovascular risk screening programmes. This included overweight or obese youth/younger adults, women with a history of gestational diabetes, and men who infrequently attend general practice. Epidemiological data indicate early onset of pre-diabetes/T2DM is increasing particularly amongst high-risk ethnic groups who generally come from populations with higher proportions of younger age groups, and for whom earlier onset of diabetes is associated with worse outcomes [65]. Those with a family history of T2DM, or who cohabit [69], or are children of women who have had gestational diabetes [70] are at higher risk for T2DM. This results in intergenerational patterns of diabetes. In families and communities where diabetes is endemic it can become normalised and ‘diabetes fatalism’ [57], where the expectation that diabetes is inevitable and is unchangeable, can become a barrier to change [71]. 

Current models of care identify individuals at risk of diabetes based on biomedical markers and rely on the individual attending general practice to be diagnosed and receive advice and support to prevent T2DM. This approach fails to make the most of the opportunity to identify clusters of people in whānau/family or communities who are also at risk of diabetes, and work with them in systematic ways with strength based approaches to improve the health of whole whānau/family or community and prevent diabetes across the entire life course [41, 61, 64, 72, 73]. Any new approaches to care need to be codesigned with the communities, and acknowledge and utilise community aspirations, strengths, knowledge, and capacity, and receive sustainable specific funding. Recent programmes developed in partnership with communities [74–81] could inform such developments. Individually focused pre-diabetes care provided within general practice would still have an important role as those with pre-diabetes frequently have other risk factors and/or comorbidities which when well managed also reduce health risks.

The current NZ guidelines impact on pre-diabetes care with the wide range of HbA1c, (41–49 mmol/mol), classifying very large numbers of people as having pre-diabetes [9]. However, a proportion of these people will never develop T2DM, and some will naturally revert to normoglycaemia. Clinicians in this study were concerned about the inability to identify and target those who most need pre-diabetes care [82, 83]. In NZ and likely in other counties, improved risk stratification is needed. In NZ, we currently lack robust long-term data [84] and tools to simply and accurately estimate the risk of progression to T2DM, associated morbidities and poorer long-term outcomes. This limits our ability facilitate pro-equity approaches to pre-diabetes care through appropriately targeted use of resources [62]. 

Pre-diabetes/T2DM is strongly associated with overweight and obesity [1] and when present, weight loss of 7% of bodyweight is key to reducing diabetes risk [43]. Neither practice routinely advised patients to lose weight, expressing reluctance to approach this contentious issue, concerns about stigmatisation, and cultural sensitivities related to obesity. Furthermore, clinicians from both practices were highly aware of some individuals limited capacity to make lifestyle changes, especially when social deprivation was a factor and this seemed to inhibit their willingness to specifically address obesity. Both these practices lacked ready access to dietetic support [61], adequate time resourcing, and possibly the skill sets for this complex work. Amongst some cultural groups specific approaches such as whānau/family strength based approaches using holistic models rather than traditional individualistic approaches based on narrow biomedical models are likely to be more effective methods to support change [71, 73, 85–88]. 

Metformin is the only glucose lowering medication recommended in NZ guidelines for pre-diabetes [35]; however, there is inadequate guidance provided and we found inconsistent prescribing patterns between practices. Notably, Metformin was prescribed more frequently in Practice B, and this may relate to clinician’s awareness of the impact of SDOH on the uptake of lifestyle and dietary changes or may relate to their having witnessed higher rates of progression to T2DM amongst the population they served. Metformin may have a greater place pre-diabetes care; however, previous research shows that the uptake of Metformin is lower in disadvantaged groups and this would need to be addressed to improve health equity [89]. 

Overall, we found that when pre-diabetes care was provided, the extent and nature of the care varied significantly. Unsurprisingly neither practice was able to provide the intensity or long-term support delivered in proven diabetes prevention programmes which typically include 26–30 contacts over 18–36 months [45, 90]. Furthermore, other than individuals’ HbA1c measures, these practices did not have the resources or incentives to evaluate the outcomes of pre-diabetes care provided overall, but instead continued ‘*plodding away’*.

In summary, our findings demonstrate complex and multi-layered issues in current provision of pre-diabetes care by general practices in NZ and highlight some possible avenues to refine and reshape this work. These recommendations are collated and summarised in Table 5.

**Table 5:** Summary of recommendations for future diabetes prevention work

1. Implement a whole of systems approach to pre-diabetes care which honours the principles of Te Tiriti o Waitangi^a^, and in addition to general practice care includes public health and social services measures. This should address the fundamental root causes of pre-diabetes and T2DM such as inequalities in SDOH, racism, food environments, and physical environments
2. Review funding systems, to ensure proactive, comprehensive equitable pre-diabetes care is incentivised and can be provided in a range of settings including general practice or community settings
2.1 Appropriately fund pre-diabetes care in general practice and other organisations, with particular emphasis on resourcing services and different disciplines and skill sets for team-based interprofessional care. Specify the skill sets and agencies required to provide comprehensive culturally appropriate lifestyle interventions and how they should work collaboratively
3. Develop the evidence base for effective and sustainable lifestyle modification particularly in relation to high-risk populations. Such approaches may best be done through a whānau ora model [91] which is integrated into care pathways and guidelines
3.1 Given the evidence, the fundamental importance of weight loss in diabetes prevention needs to be emphasised in diabetes prevention services; however, this must be done in a culturally tailored manner
3.1.1 Implement evidence-based measures to support weight loss including use of dieticians, and culturally adapted community-led, [92] whānau/group or possible commercial programmes
3.2 Partner with communities affected by high prevalence of T2DM (such as groups related to ethnicity, geographic region, socio-economic status, community or intergenerational patterns of diabetes) to develop and employ new models of diabetes prevention which are community/whānau focused, culturally congruent and target multigenerational patterns of diabetes
3.3 Research the outcomes of current and new models of care
4. Refine national guidelines for pre-diabetes care
4.1 Develop simple tools to risk stratify those with pre-diabetes, so that higher risk groups can be more intensively targeted, and resources used wisely
4.2 Emphasise the importance of pre-diabetes care in management guidelines. Ensure the guidelines:
4.2.1 include social deprivation in the list of risk factors for T2DM, so this is highlighted, and those experiencing deprivation are appropriately screened
4.2.2 develop separate pre-diabetes treatment algorithms which:
4.2.2.1 specify recommended treatment intensity, treatment escalation and frequency of monitoring which are linked to level of risk
4.2.2.2 clarify when and in what groups metformin should be prescribed
4.2.2.3 incorporate appropriate guidance for assessment and management of other risk factors or co-morbidities
4.2.3 acknowledge that deprivation makes attending appointments and adopting evidence-based guidance more challenging and integrate into guidelines how this can be addressed

^a^Te Tiriti o Waitangi refers to the Māori version of the founding document reflecting the principles of agreement between the British and Māori to establish a nation state and build a government in New Zealand

### Strengths and limitations

This is the first study to describe the routine methods used to detect and manage pre-diabetes care in general practice in NZ. The multiple case study design enabled an in-depth study of two diverse practices revealing similarities and contrasting concerns. Further work including Māori and Pacific researchers and purposefully including Māori, Pacific and rural health care providers would build on these cases and may confirm these findings and/or identify different facilitators and barriers to pre-diabetes care, especially if different models for funding and care delivery are used. The inclusion of two iterations of focus groups to verify and collect additional data and the clinical notes review ensured a rigorous approach and enabled triangulation of data. It confirmed general practices have other pressing concerns related to multimorbidity/acute presentations and these are prioritised ahead of pre-diabetes care. Both practices verified that interactions related to pre-diabetes are infrequently documented. It is possible that the focus groups may not have elicited the specifics of what occurs in clinical interactions related to pre-diabetes care [61] and other methods of data collection such as direct observation or videoed consultations might fill these gaps. More broadly, while NZ general practices have unique characteristics, many similar issues may exist in international contexts, and particularly where diabetes disparities are found related to ethnicity and SDOH.

## Conclusions

This study of two diverse general practices in NZ identified multiple influences on pre-diabetes care provision. Health system policy and funding mechanisms dis-incentivise pre-diabetes care. The numbers currently classified as having pre-diabetes, who have varying but unspecified levels of risk, increase workloads to the point where it is not possible to systematically deliver pre-diabetes care along with other general practice work. Social deprivation independently increases diabetes risk and decreases the ability of individuals to access and respond to care. This creates a double jeopardy for ethnic groups who experience higher rates of T2DM. However, this is not acknowledged or addressed in current funding, policies and guidelines, and consequently places those who provide care to these populations under enormous pressure. Inconsistencies and gaps in evidence, guidelines, screening practices and individual care were also found. Together these findings highlight multiple avenues to improve pre-diabetes work to prevent the development of T2DM more effectively and concurrently reduce pre-diabetes/T2DM related health inequities. Any redesign of this work must be responsive to cultural and social contexts including the SDOH and requires multisectoral approaches including public health, social service, general practice community and indigenous organisations.

## Supplementary Information

**Additional file 1: Supplementary Table 1.** Clinical notes data coding, extraction, and presentation procedures. **Supplementary Figure 1A.** Case review example 1. **Supplementary Figure 1B.** Case review example 2.

## Data Availability

The datasets generated and analysed during the current study are not publicly available however any requests for materials should be directed to Christine.Barthow@otago.ac.nz.

## Abbreviations

BMI: Body mass index
GPs: General practitioners
HbA1c: Glycated haemoglobin
HIP: Health improvement practitioners
SDOH: Social determinants of health
T2DM: Type 2 diabetes mellitus
NZ: Aotearoa/New Zealand
VLCA: Very low-cost access

## References

[1] Zheng Y, Ley SH, Hu FB (2018). Global aetiology and epidemiology of type 2 diabetes mellitus and its complications. Nat Rev Endocrinol.

[2] Lin X, Xu Y, Pan X, Xu J, Ding Y, Sun X (2020). Global, regional, and national burden and trend of diabetes in 195 countries and territories: an analysis from 1990 to 2025. Sci Rep.

[3] International Diabetes Federation. IDF diabetes atlas. 10th ed. Brussels, Belgium: International Diabetes Federation; 2021 [cited 2022 Feb 1]. Available from: www.diabetesatlas.org

[4] Zimmet PZ (2017). Diabetes and its drivers: the largest epidemic in human history?. Clin Diabetes Endocrinol.

[5] Hill-Briggs F, Adler NE, Berkowitz SA, Chin MH, Gary-Webb TL, Navas-Acien A (2021). Social determinants of health and diabetes: a scientific review. Diab Care..

[6] Rett K, Gottwald-Hostalek U (2019). Understanding prediabetes: definition, prevalence, burden and treatment options for an emerging disease. Curr Med Res Opin..

[7] Tabák AG, Herder C, Rathmann W, Brunner EJ, Kivimäki M (2012). Prediabetes: a high-risk state for diabetes development. Lancet.

[8] Mainous AG, Tanner RJ, Baker R, Zayas CE, Harle CA. Prevalence of prediabetes in England from 2003 to 2011: Population-based, cross-sectional study. BMJ Open. 2014;4(6). 10.1136/bmjopen-2014-005002 PMC4054625 24913327

[9] Coppell KJ, Mann JI, Williams SM, Jo E, Drury PL, Miller J (2013). Prevalence of diagnosed and undiagnosed diabetes and prediabetes in New Zealand: findings from the 2008/09 adult nutrition survey. N Z Med J.

[10] Knowler WC, Barrett-Connor E, Fowler SE, Hamman RF, Lachin JM, Walker EA (2002). Reduction in the incidence of type 2 diabetes with lifestyle intervention or metformin. N Engl J Med..

[11] Tuomilehto J, Lindström J, Eriksson J, Valle T, Hämäläinen H, Ilanne-Parikka P (2001). Prevention of type 2 diabetes mellitus by changes in lifestyle among subjects with impaired glucose tolerance. N Engl J Med.

[12] Pan X, Li G, Hu Y, Wang J, Yang W, An Z (1997). Effects of diet and exercise in preventing NIDDM in people with impaired glucose tolerance. Diabetes Care.

[13] Hamman RF, Wing RR, Edelstein SL, Lachin JM, Bray GA, Delahanty L (2006). Effect of weight loss with lifestyle intervention on risk of diabetes. Diabetes Care..

[14] Kriska AM, Rockette-Wagner B, Edelstein SL, Bray GA, Delahanty LM, Hoskin MA (2021). The impact of physical activity on the prevention of type 2 diabetes: evidence and lessons learned from the diabetes prevention program, a long-standing clinical trial incorporating subjective and objective activity measures. Diabetes Care..

[15] Aroda VR, Knowler WC, Crandall JP, Perreault L, Edelstein SL, Jeffries SL (2017). Metformin for diabetes prevention: insights gained from the diabetes prevention program/diabetes prevention program outcomes study. Diabetologia.

[16] Knowler WC, Edelstein SL, Goldberg RB, Ackermann RT, Crandall JP, Florez JC (2015). HbA1c as a predictor of diabetes and as an outcome in the diabetes prevention program: a randomized clinical trial. Diabetes Care..

[17] Diabetes Prevention Program Research Group (2019). Long-term effects of metformin on diabetes prevention: identification of subgroups that benefited most in the diabetes prevention program and diabetes prevention program outcomes study. Diabetes Care.

[18] Madsen KS, Chi Y, Metzendorf M-I, Richter B, Hemmingsen B. Metformin for prevention or delay of type 2 diabetes mellitus and its associated complications in persons at increased risk for the development of type 2 diabetes mellitus. Cochrane Database Syst Rev. 2019;(12). Available from: http://doi.wiley.com/10.1002/14651858.CD008558.pub2 10.1002/14651858.CD008558.pub2 PMC6889926 31794067

[19] Valabhji J, Barron E, Bradley D, Bakhai C, Fagg J, O’Neill S (2020). Early Outcomes From the English National Health Service Diabetes Prevention Programme. Diabetes Care..

[20] Dunbar JA (2017). Diabetes prevention in Australia: 10 years results and experience. Diabetes Metab J.

[21] World Health Organization. Regional Office for the Western Pacific. New Zealand Health system review. Vol. 4. WHO Regional Office for the Western Pacific; 2014. Available from: https://apps.who.int/iris/handle/10665/207738

[22] Irurzun-Lopez M, Jeffreys M, Cumming J (2021). The enrolment gap: who is not enrolling with primary health organizations in Aotearoa New Zealand and what are the implications? An exploration of 2015–2019 administrative data. Int J Equity Health..

[23] Thomson M (2019). Who had access to doctors before and after new universal capitated subsidies in New Zealand?. Health Policy (New York).

[24] Corscadden L, Levesque JF, Lewis V, Strumpf E, Breton M, Russell G (2018). Factors associated with multiple barriers to access to primary care: An international analysis. Int J Equity Health.

[25] Ministry of Health. Strategic Intentions 2021 to 2025. Wellington; 2021. Available from: https://www.health.govt.nz/system/files/documents/publications/strategic_intentions_2021-2025-withcover_9_dec.pdf

[26] Goodyear-Smith F, Ashton T (2019). New Zealand health system: universalism struggles with persisting inequities. Lancet.

[27] Yin R (2018). Case study research and applications: design and methods.

[28] Harrison H, Birks M, Franklin R, Mills J (2017). Case study research: foundations and methodological orientations. Forum Qual Sozialforsch / Forum Qual Soc Res..

[29] Fàbregues S, Fetters MD (2019). Fundamentals of case study research in family medicine and community health. Fam Med Community Heal..

[30] Trajkovski S, Schmied V, Vickers M, Jackson D (2013). Using appreciative inquiry to transform health care. Contemp Nurse.

[31] Cresswell J, Poth C (2018). Qualitative research design choosing among five approaches.

[32] Vassar M, Matthew H (2013). The retrospective chart review: important methodological considerations. J Educ Eval Health Prof..

[33] Hsieh H-F, Shannon SE (2005). Three approaches to qualitative content analysis. Qual Health Res..

[34] Braun V, Clarke V (2006). Using thematic analysis in psychology. Qual Res Psychol..

[35] NZSSD, Ministry of Health. Type 2 Diabetes Management Guidance. 2021 [cited 2021 Jun 24]. Available from: https://t2dm.nzssd.org.nz/Home.html#carousel_1d48

[36] Ministry of Health. Cardiovascular disease risk assessment and management for primary care. Wellington: Ministry of Health; 2018. Available from: https://www.health.govt.nz/system/files/documents/publications/cvd-risk-assessment-and-management-for-primary-care-v2.pdf

[37] New Zealand Ministry of Health. Green prescriptions. 2021 [cited 2021 Sep 23]. Available from: https://www.health.govt.nz/our-work/preventative-health-wellness/physical-activity/green-prescriptions

[38] TePou. Health improvement practitioners in New Zealand. 2022 [cited 2022 Jul 19]. Available from: https://www.tepou.co.nz/initiatives/integrated-primary-mental-health-and-addiction/health-improvement-practitioners-in-new-zealand

[39] Ministry of Health. Annual Data Explorer 2019/20: New Zealand health survey. 2020 [cited 2021 Nov 10]. Available from: https://minhealthnz.shinyapps.io/nz-health-survey-2019-20-annual-data-explorer/

[40] Kyrou I, Tsigos C, Mavrogianni C, Cardon G, Van Stappen V, Latomme J (2020). Sociodemographic and lifestyle-related risk factors for identifying vulnerable groups for type 2 diabetes: a narrative review with emphasis on data from Europe. BMC Endocr Disord.

[41] Messina J, Campbell S, Morris R, Eyles E, Sanders C (2017). A narrative systematic review of factors affecting diabetes prevention in primary care settings. PLoS One..

[42] Stokes T, Tumilty E, Doolan-Noble F, Gauld R (2017). Multimorbidity, clinical decision making and health care delivery in New Zealand primary care: a qualitative study. BMC Fam Pract..

[43] American Diabetes Association. 3 (2021). Prevention or delay of type 2 diabetes: standards of medical care in diabetes -2021. Diabetes Care..

[44] Thomas C, Sadler S, Breeze P, Squires H, Gillett M, Brennan A (2017). Assessing the potential return on investment of the proposed UK NHS diabetes prevention programme in different population subgroups: an economic evaluation. BMJ Open..

[45] Ackermann RT, O’Brien MJ (2020). Evidence and challenges for translation and population impact of the diabetes prevention program. Curr Diab Rep..

[46] Stokes T, Azam M, Noble FD (2018). Multimorbidity in Māori and Pacific patients: Cross-sectional study in a Dunedin general practice. J Prim Health Care.

[47] Gurney J, Stanley J, Sarfati D. The inequity of morbidity: disparities in the prevalence of morbidity between ethnic groups in New Zealand. J Comorbidity. 2020. 10:2235042X2097116. Available from: http://journals.sagepub.com/doi/10.1177/1049732305276687 10.1177/2235042X20971168 PMC7658519 33224894

[48] Stanley J, Semper K, Millar E, Sarfati D (2018). Epidemiology of multimorbidity in New Zealand: a cross-sectional study using national-level hospital and pharmaceutical data. BMJ Open..

[49] Te Karu L, Harwood M, Bryant L, Kenealy T, Arroll B (2021). Compounding inequity: a qualitative study of gout management in an urban marae clinic in Auckland. J Prim Health Care..

[50] Castle L, Bradshaw M, Patel-Campbell T, Holmes M, McEvoy J (2020). Shortfalls of funding for general practice in deprived areas. Br J Gen Pract..

[51] Maple-Brown LJ, Hampton D (2020). Indigenous cultures in countries with similar colonisation histories share the challenge of intergenerational diabetes. Lancet Glob Heal.

[52] Voaklander B, Rowe S, Sanni O, Campbell S, Eurich D, Ospina MB (2020). Prevalence of diabetes in pregnancy among Indigenous women in Australia, Canada, New Zealand, and the USA: a systematic review and meta-analysis. Lancet Glob Heal.

[53] Markwick A, Ansari Z, Sullivan M, Parsons L, McNeil J (2014). Inequalities in the social determinants of health of Aboriginal and Torres Strait Islander People: a cross-sectional population-based study in the Australian state of Victoria. Int J Equity Health..

[54] Health Quality & Safety Commission New Zealand. A window on the quality of Aotearoa New Zealand’s health care 2019 – a view on Māori health equity. 2019. Available from: https://www.hqsc.govt.nz/assets/Health-Quality-Evaluation/PR/Window_2019_web_final.pdf

[55] Ogunwole SM, Golden SH (2021). Social determinants of health and structural inequities - root causes of diabetes disparities. Diabetes Care..

[56] Vanstone M, Rewegan A, Brundisini F, Giacomini M, Kandasamy S, DeJean D (2017). Diet modification challenges faced by marginalized and nonmarginalized adults with type 2 diabetes: a systematic review and qualitative meta-synthesis. Chronic Illn..

[57] Power T, Kelly R, Usher K, East L, Travaglia J, Robertson H (2020). Living with diabetes and disadvantage: a qualitative, geographical case study. J Clin Nurs..

[58] Higgins T, Larson E, Schnall R (2017). Unraveling the meaning of patient engagement: a concept analysis. Patient Educ Couns.

[59] Spencer Bonilla G, Rodriguez-Gutierrez R, Montori VM (2016). What we don’t talk about when we talk about preventing type 2 diabetes - addressing socioeconomic disadvantage. JAMA Intern Med..

[60] Smith CJ, McNaughton DA, Meyer SB (2021). Implications for clients when nurses view weight as main cause of type 2 diabetes in primary care. Aust J Prim Health..

[61] Somerville M, Ball L, Sierra-Silvestre E, Williams LT (2019). Understanding the knowledge, attitudes and practices of providing and receiving nutrition care for prediabetes: an integrative review. Aust J Prim Health..

[62] Starck CS, Blumfield M, Keighley T, Marshall S, Petocz P, Inan-Eroglu E (2021). Nutrient dense, low-cost foods can improve the affordability and quality of the New Zealand diet - a substitution modeling study. Int J Environ Res Public Health..

[63] Gruss SM, Nhim K, Gregg E, Bell M, Luman E, Albright A. Public Health Approaches to Type 2 Diabetes Prevention: the US National Diabetes Prevention Program and Beyond. Curr Diab Rep. 2019;19(9). 10.1007/s11892-019-1200-z PMC6682852 31385061

[64] Timpel P, Harst L, Reifegerste D, Weihrauch-Blüher S, Schwarz PEH (2019). What should governments be doing to prevent diabetes throughout the life course?. Diabetologia..

[65] Price Waterhouse Cooper. Report on the economic and social cost of type 2 diabetes. Healthier Lives; 2021 [cited 2021 Jul 19]. Available from: https://healthierlives.co.nz/report-on-the-economic-and-social-cost-of-type-2-diabetes/

[66] Tawfiq E, Bradbury KE, Ni Mhurchu C (2021). Healthiness of foods and non-alcoholic beverages according to store type: a population-based study of household food and drink purchases in New Zealand. SSM Popul Heal..

[67] McKerchar C, Smith M, Gage R, Williman J, Abel G, Lacey C (2020). Kids in a candy store: an objective analysis of children’s interactions with food in convenience stores. Nutrients..

[68] Blakely T, Cleghorn C, Mizdrak A, Waterlander W, Nghiem N, Swinburn B (2020). The effect of food taxes and subsidies on population health and health costs: a modelling study. Lancet Public Heal.

[69] Goyal A, Gupta Y, Kalaivani M, Sankar MJ, Kachhawa G, Bhatla N (2019). Concordance of glycaemic and cardiometabolic traits between Indian women with history of gestational diabetes mellitus and their spouses: an opportunity to target the household. Diabetologia..

[70] Shou C, Wei Y-M, Wang C, Yang H-X (2019). Updates in long-term maternal and fetal adverse effects of gestational diabetes mellitus. Matern Med..

[71] Tane T, Selak V, Hawkins K, Lata V, Murray J, Nicholls D (2021). Māori and Pacific peoples’ experiences of a Māori-led diabetes programme. N Z Med J.

[72] Abel SL, Whitehead LC, Tipene-Leach DC, Coppell KJ (2021). Proximal and distal influences on dietary change among a diverse group with prediabetes participating in a pragmatic, primary care nurse-led intervention: a qualitative study. Public Health Nutr..

[73] Glover M, Wong SF, Taylor RW, Derraik JGB, Fa’alili-Fidow J, Morton SM (2019). The complexity of food provisioning decisions by Māori caregivers to ensure the happiness and health of their children. Nutrients..

[74] Ni Mhurchu C, Te Morenga L, Tupai-Firestone R, Grey J, Jiang Y, Jull A (2019). A co-designed mHealth programme to support healthy lifestyles in Māori and Pasifika peoples in New Zealand (OL@-OR@): a cluster-randomised controlled trial. Lancet Digit Heal.

[75] Verbiest M, Borrell S, Dalhousie S, Tupa’i-Firestone R, Funaki T, Goodwin D (2018). A co-designed, culturally-tailored mHealth tool to support healthy lifestyles in Māori and Pasifika communities in New Zealand: protocol for a cluster randomized controlled trial. JMIR Res Protoc..

[76] Verbiest MEAA, Corrigan C, Dalhousie S, Firestone R, Funaki T, Goodwin D (2019). Using codesign to develop a culturally tailored, behavior change mHealth intervention for indigenous and other priority communities: a case study in New Zealand. Transl Behav Med..

[77] Firestone R, Faeamani G, Okiakama E, Funaki T, Henry A, Prapaveissis D (2021). Pasifika prediabetes youth empowerment programme: evaluating a co-designed community-based intervention from a participants’ perspective. Kotuitui.

[78] Tupai-Firestone R, Matheson A, Prapavessis D, Hamara M, Kaholokula K, Tuisano H (2018). Pasifika youth empowerment programme: a potential public health approach in tackling obesity-health related issues. Altern An Int J Indig Peoples..

[79] Firestone R, Cheng S, Dalhousie S, Hughes E, Funaki T, Henry A (2020). Exploring Pasifika wellbeing: findings from a large cluster randomised controlled trial of a mobile health intervention programme. N Z Med J.

[80] Selak V, Stewart T, Jiang Y, Reid J, Tane T, Carswell P (2018). Indigenous health worker support for patients with poorly controlled type 2 diabetes: study protocol for a cluster randomised controlled trial of the Mana Tū programme. BMJ Open..

[81] Harwood M, Tane T, Broome L, Carswell P, Selak V, Reid J (2018). Mana Tū: a whānau ora approach to type 2 diabetes. N Z Med J.

[82] Sampson M, Elwell-Sutton T, Bachmann MO, Clark A, Dhatariya KK, Ferns C (2018). Discordance in glycemic categories and regression to normality at baseline in 10,000 people in a type 2 diabetes prevention trial. Sci Rep.

[83] McKinlay E, Hilder J, Hood F, Morgan S, Barthow C, Gray B (2022). Uncertainty and certainty: perceptions and experiences of prediabetes in New Zealand primary care – a qualitative study. J Prim Health Care..

[84] Teng A, Blakely T, Scott N, Jansen R, Masters-Awatere B, Krebs J (2019). What protects against pre-diabetes progressing to diabetes? Observational study of integrated health and social data. Diabetes Res Clin Pract.

[85] Wild CEK, Rawiri NT, Willing EJ, Hofman PL, Anderson YC (2021). What affects programme engagement for Māori families? A qualitative study of a family-based, multidisciplinary healthy lifestyle programme for children and adolescents. J Paediatr Child Health..

[86] Bell R, Smith C, Hale L, Kira G, Tumilty S (2017). Understanding obesity in the context of an Indigenous population—A qualitative study. Obes Res Clin Pract.

[87] Eyles H, Mhurchu CN, Wharemate L, Funaki-Tahifote M, Lanumata T, Rodgers A (2009). Developing nutrition education resources for a multi-ethnic population in New Zealand. Health Educ Res.

[88] Murphy E, McAuley KA, Bell D, McLay RT, Chisholm A, Hurley R (2003). A new approach to design and implement a lifestyle intervention programme to prevent type 2 diabetes in New Zealand Maori. Asia Pac J Clin Nutr..

[89] Chepulis L, Mayo C, Morison B, Keenan R, Lao C, Paul R (2020). Metformin adherence in patients with type 2 diabetes and its association with glycated haemoglobin levels. J Prim Health Care.

[90] Research DPP (DPP) Group (2002). The diabetes prevention program (DPP): description of lifestyle intervention. Diabetes Care..

[91] New Zealand Ministry of Health. Whānau Ora programme. 2018 [cited 2021 Sep 23]. Available from: https://www.health.govt.nz/our-work/populations/maori-health/whanau-ora-programme

[92] Letele D. Buttabean motivation. [cited 2021 Sep 27]. Available from: https://www.thebbmprogram.com/about-dave-letele/

