# Supplementary material for: A multiple case study of pre-diabetes care undertaken by general practice in Aotearoa/New Zealand: de-incentivised and de-prioritised work
Source: BMC Prim Care. 2023 Apr 29;24:109. doi: 10.1186/s12875-023-02053-1 (PMC10147904; doi:10.1186/s12875-023-02053-1)
Supplement: Supplementary file 1 — Additional file 1: Supplementary Table 1. Clinical notes data coding, extraction, and presentation procedures. Supplementary Figure 1A. Case review example 1. Supplementary Figure 1B. Case review example 2. [file 12875_2023_2053_MOESM1_ESM.pdf]

Supplementary files: Barthow, et al 2023: A multiple case study of pre-diabetes care undertaken by general practice in Aotearoa/New Zealand: de-incentivised and de-prioritised work

Supplementary Table 1 Clinical notes data coding, extraction, and presentation procedures

For each set of notes:

1. A summary profile of each patient was developed
2. Serial HbA1c and body mass index (BMI) data were extracted
3. Notes were coded and data extracted in a dichotomous manner in six-month time frames and according to provider type for the presence or absence of
   a. four key evidence-based diabetes prevention recommendations - weight reduction; physical activity, dietary modification, and smoking cessation
   b. other health promotion activities including blood pressure monitoring; cardiovascular risk screening; immunisations; and cervical screening
4. The frequency of face-to-face visits were recorded according to six-month timeframes and provider type (GP, nurse, health support worker)
5. Where diabetes prevention activities occurred a summary of the reason for the visit was recorded and the specific diabetes prevention notes extracted and coded by provider type
6. A composite clinical note summary was created using Excel displaying the data in a sequential and graphic format with the accompanying profile and clinical note data
7. Summary findings from the clinical notes were generated and included in the summary

Legend and coding notes for Supplementary Figure 1A and 1B

Symbol	Serial HbA1c and BMI graph
	Serial HbA1c
	Serial BMI
	Patient notified HbA1c result
	**Provider colour codes**
	General practitioner
	Nurse
	Health support worker
	**Pre-diabetes assessment and management**
	Weight
	Physical activity
	Diet
	Smoking Cessation
	Lifestyle intervention - unspecified
	Metformin discussed/prescribed
	**Health screening and protection activities**
	Immunisation
	Cervical screening
	Cardiovascular risk screening
	Blood pressure measured

Notes

1. Coding does not distinguish between clinician note to self-versus direct discussion with patient – see clinical notes for details
2. Dietary advice and physical activity recommendations were coded under pre-diabetes activities regardless of the rationale for recommendation - e.g., if physical activity was recommended for mental health, it was still coded as beneficial for pre-diabetes
3. For serial pre-diabetes assessment & management and other health screening and health protection activities data icons reflect the presence of an activity or mention in the notes but not the frequency of occurrence

Case ID	Practice A Case F
Age range	55-60
Gender	Female
BMI range	Overweight
Ethnicity	Cook Island Maori
Smoking Status	Current smoker
Family History T2DM	Yes
Co-morbidities	Musculosketal
Regular medications	3
Year diagnosed	2012
Dashboard	Diabetes screening , Conditions of interest, not on recall
Referrals offered	Surgical

Summary

- Reluctant to stop smoking
- No detail of dietary / physical activity discussion (multiple refusals smoking cessation support) focus appears target driven
- GP management
- Hard to interpret when notes were direct discussion with patient vs comment to alert staff/self for future appointments

Clinical notes

Feb 2012 GP	Patient advised of results as per borderline diabetic P:needs to get wt close to 70kg, annual HbA1c
June 2015 GP	Presentation: smear<br>Still smoking , less than she used to, about 5/d, not interested in help stopping…<br>O:\we 83.4 \bp 154/86<br>….<br>Assessment of finger<br>……<br>re start statin …plan re finger….<br>Watch wt as going up & HbA1c isq but wt gain is biggest risk to going on to get niddm
May 2018 GP	[Ibx:HbA1c 45-stable] Message left for patient to call back re lipids & CVR risk, ultimately more value in stopping smoking than taking statin (via voice mail)
May 2019 GP	Text from Gp to get annual prediabetes bloods done

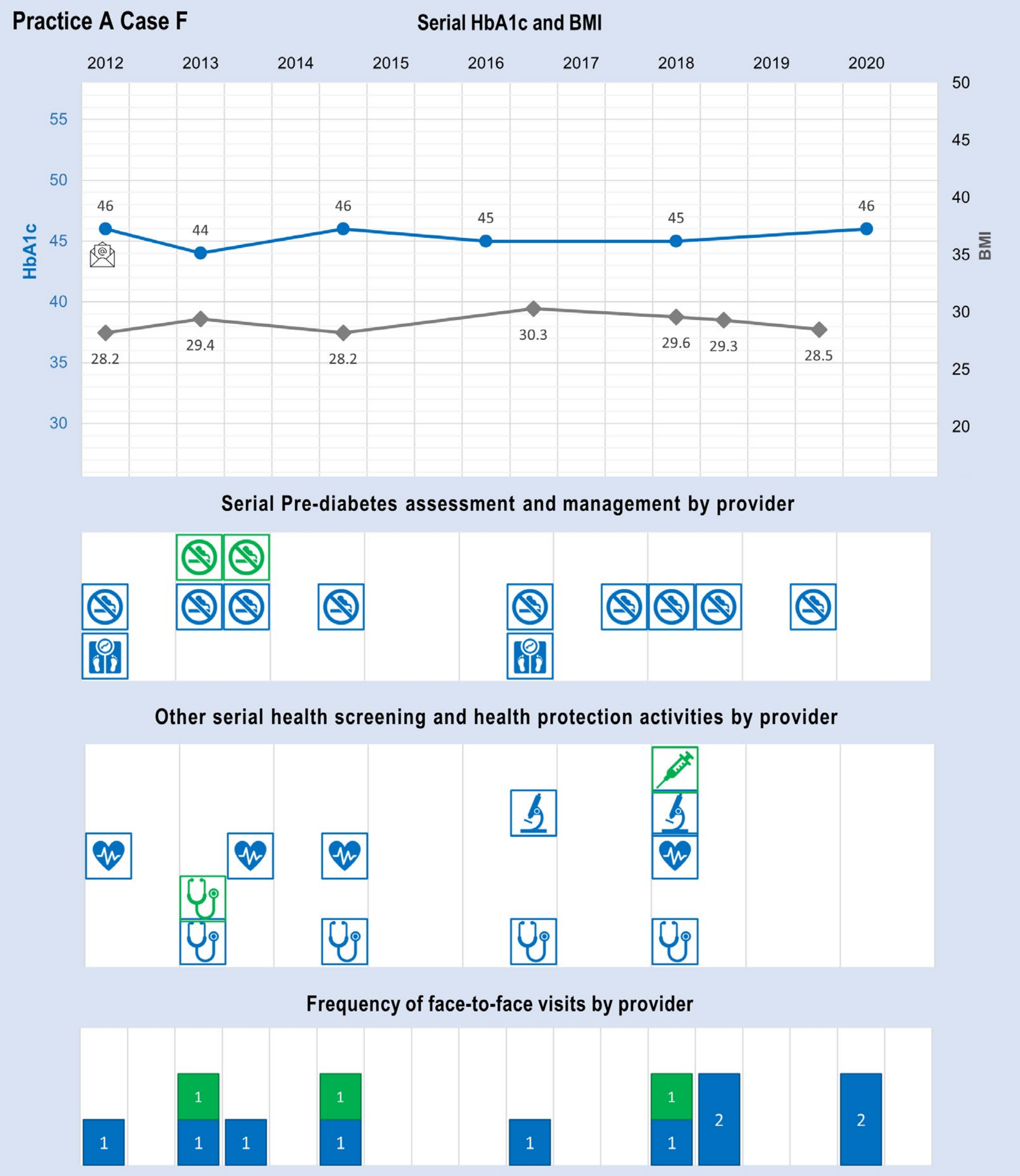

Supplementary Figure 1A Case review example 1

Case ID	Practice B Case E	Summary
Age range	60-65	• Very complex case, multiple walk-in appointments. Significant other issues, impinging on the ability to work with pre-diabetes<br>• Not clear if early HbA1c results discussed<br>• Increasing BMI reflected in rising HbA1c<br>• Dietary and physical activity advice interwoven as part of other health interventions
Gender	Female	
BMI range	Normal - obese	
Ethnicity	Māori	
Smoking status	Ex-smoker	
Family History T2DM	Yes	
Co-morbidities	Mental health	
Regular medications	1	
Year diagnosed	Unclear - pre-2013?	
Dashboard	Recalls HbA1c for pre-diabetes and CVD	
Referrals offered	Surgical, neurological, mental health, HIP (mental health)	

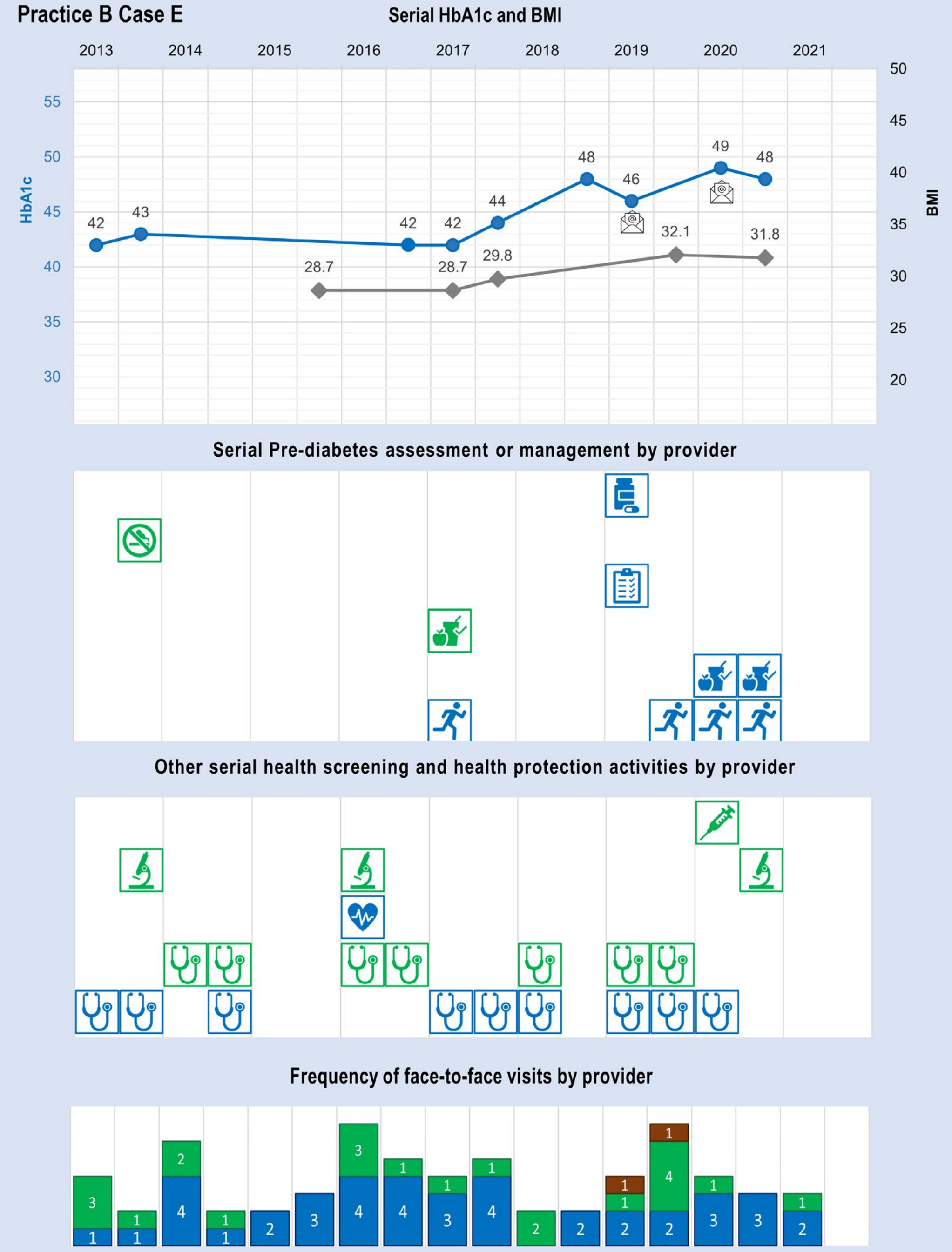

Clinical notes	
Jul 2013 Ns	Presenting issue: Cervical smear …currently smoking again after 2 years being a non-smoker cessation advise given, at this stage not wanting to quit aware she can come back for treatment…
May 2014 Ns	Voicemail to remind of annual diabetes review
April 2017 GP	Presenting issue: multiple vague symptoms …imp anxiety …encouraged to do exercises and maintain activity…
April 2017 Ns	Presenting issue: BP check request bt – concerned about anemia .. Plan) bt, eat well-lots of veges and green veges, red meat, fluids++,
Jan 2019 GP	Presenting issue: multiple complaints ….discussed results of last BT Dec 2018--Hb/iron levels n, HbA1c 48--see nurse for dietary advice….
May 2019 GP	Presenting issue: for review blood tests, cough for 3 days discussed prediabetes not keen on taking metformin yet but will try lifestyle changes first….
Nov 2019 GP	Presenting issue: sore throat, headaches, anxiety …30 mins exercise 3x/wk….
May 2020 GP	Presenting issue: Fu blood results – Ph consult Discussed HbA1c getting very close to diabetes level. Suggest she get back to some healthy eating and increase her exercise aim for 30mins of walking/d, doesn't have to be all at once, can break it up…
2020 GP	Presenting issue: multiple …Dx: Health ed. - diet (6799.00), Dx: Health ed. - exercise (6798.00)…

Supplementary Figure 1B Case review example 2
